# Outcome of elective cemiplimab discontinuation in locally advanced or metastatic cutaneous squamous cell carcinoma patients achieving a complete remission

**DOI:** 10.3389/fonc.2026.1655448

**Published:** 2026-02-05

**Authors:** Tina Fung, Wolfram Samlowski

**Affiliations:** 1 Kirk Kerkorian School of Medicine at UNLV, Las Vegas, NV, United States; 2 Nevada Oncology Specialists, Las Vegas, NV, United States; 3 Department of Internal Medicine, Kirk Kerkorian School of Medicine at UNLV, University of Nevada, Las Vegas (UNLV), Las Vegas, NV, United States

**Keywords:** checkpoint inhibitor, elective treatment discontinuation, keratinocyte carcinoma, PD-1 antibody, skin cancer

## Abstract

**Introduction:**

Cemiplimab treatment of patients with locally advanced or metastatic cutaneous squamous cell carcinoma induced rapid and deep tumor regressions. The majority of our cemiplimab-treated patients achieved a complete remission. It was not clear how long cemiplimab therapy should be continued in these patients, to prevent a subsequent relapse.

**Materials and Methods:**

We identified cutaneous squamous cell carcinoma patients who were treated with cemiplimab via a computer database search. Individual patient records were then reviewed to identify patients who had achieved a radiographically or pathologically confirmed complete remission. Following elective treatment discontinuation based on our institutional standard, the outcomes of these patients were analyzed.

**Results:**

Complete remissions were achieved in 15 out of 21 patients (71.4%) following cemiplimab treatment. The median treatment duration was only 5.3 ± 3.7 months (or 8.0 ± 3.6 doses of cemiplimab). With a median follow-up of 48.5 months following elective treatment discontinuation, only one of 15 patients experienced a delayed relapse. The other 14 patients have remained in a durable complete remission.

**Discussion:**

Our retrospective data review demonstrated that early elective cemiplimab discontinuation was both feasible, and safe. There was a low risk of relapse if patients achieve a radiologically or pathologically documented complete remission. We believe that decreases in the duration of cemiplimab treatment has the potential to reduce the risk of delayed checkpoint inhibitor toxicity, as well as decreasing treatment costs. Confirmation of our encouraging findings in a prospective clinical trial is recommended.

## 1 Introduction

Cutaneous squamous cell carcinoma (CSCC) is the second most prevalent skin cancer in the United States, with an estimated annual incidence exceeding 1,000,000 cases per year. However, precise numbers of patients affected are hard to determine as CSCC is not tracked in national cancer registries such as NCI’s Surveillance, Epidemiology, and End Results (SEER) database (1). It has also become apparent that a small percentage (3-7%) of patients develop a much more aggressive clinical course, which may progress to locally advanced or metastatic disease (2–7). For instance, a 2012 estimate suggested that between 5,604 and 12,572 patients in the United States developed metastatic CSCC, resulting in 3,932-8,791 deaths (3).

Recent advances in medical therapy have transformed the management of locally advanced or metastatic CSCC, particularly with the introduction of immune checkpoint inhibitors (ICI), such as cemiplimab and pembrolizumab (8, 9). Clinical trials demonstrated that these PD-1-directed monoclonal antibodies can mediate rapid and deep tumor responses in a significant percentage of patients, including durable progression-free survival (8–11). Complete responses can be achieved even in patients who have progressed after prior surgery or radiation (12).

In our experience, a high percentage (61.1%) of patients treated with cemiplimab therapy achieved clinical or pathologic complete remission (13). However, the optimal treatment duration required to maintain these remissions before elective treatment discontinuation can safely be considered remains uncertain. In clinical trials of cemiplimab and pembrolizumab, these patients were often treated for an arbitrary length of time (54–96 weeks) in the absence of major immune related adverse events (IRAE) (8, 14). Currently, there is no standardized guideline for the discontinuation of cemiplimab treatment and prolonged therapy may elevate the risk of developing delayed or chronic autoimmune toxicities (15, 16), as well as imposing a financial burden on patients and payers (17, 18). Additionally, extended treatment durations may adversely affect patients’ quality of life (19). It should be noted that checkpoint inhibitor discontinuation strategies in patients achieving a clinical remission have been proposed in a number of cancer types, including melanoma and CSCC (20–23). In this study, we present results from our institutional protocol for elective treatment discontinuation of ICI treatment. Although this approach was originally developed in melanoma patients (20), this strategy appears applicable to multiple other types of ICI treated cancers (21). We report on the specific application of this approach to a population of patients with locally advanced or metastatic CSCC patients treated with cemiplimab.

## 2 Materials and methods

### 2.1 Patient ascertainment

Potential subjects were identified from a search of a Health Information Portability and Accessibility Act (HIPAA) compliant iKnowMed database (IKM-G2, McKesson, Woodlands TX) for patients treated with cemiplimab by a single oncologist (WS). Individual medical records were then individually accessed, and relevant data were extracted into a password-protected Microsoft Excel spreadsheet (v16.86, Redmond, WA).

To be eligible for analysis, patients were required to have a biopsy confirmed diagnosis of cutaneous squamous cell carcinoma (CSCC). All patients were required to have received at least two doses of cemiplimab. Those with mixed squamous and basal cell carcinoma or who received cemiplimab for other indications (e.g., basal cell carcinoma or lung cancer) were excluded from analysis. Patients who received radiotherapy in addition to cemiplimab were also excluded.

Each patient was assigned a unique patient number (UPN) for anonymity. Patient demographics (age at therapy initiation, gender, race), comorbidities, and potential causes for immunosuppression were recorded. Tumor characteristics, including primary site, prior treatments (surgery, radiotherapy, or other therapies), and disease stage (locally advanced or metastatic), were documented. Metastatic sites were also noted. Following data extraction, the spreadsheet was deidentified. This study design was reviewed by the chair of the Western IRB and granted an exemption from full IRB review.

### 2.2 Treatment regimens

All patients were treated with cemiplimab at a fixed dose of 350 mg every three weeks, intravenously. Key treatment details, including the start date, duration, total number of doses, and any cemiplimab-related toxicities, were documented. Some patients experienced temporary treatment interruptions due to insurance changes, comorbid illnesses, or loss to follow-up. For these patients, treatment duration was calculated from the treatment restart date.

### 2.3 Treatment response evaluation

Objective response to cemiplimab was assessed quantitatively whenever possible using RECIST 1.1 criteria (24), with lesions measured by computerized tomography (CT), magnetic resonance imaging (MRI), or positron emission tomography (PET)-CT imaging. Complete response (CR) required complete resolution of all lesions, partial response (PR) required a >30% reduction in lesion size, progressive disease (PD) required a >20% increase of existing lesions or new metastases, and stable disease (SD) met none of these criteria. For superficial cutaneous lesions not measurable by imaging, semi-quantitative skin examinations were performed, with CR defined by disappearance of raised tumor margins and skin healing. New primary CSCCs at remote sites were documented separately. Data collection concluded June 8, 2024.

### 2.4 Elective treatment discontinuation strategy

**Figure 1:**
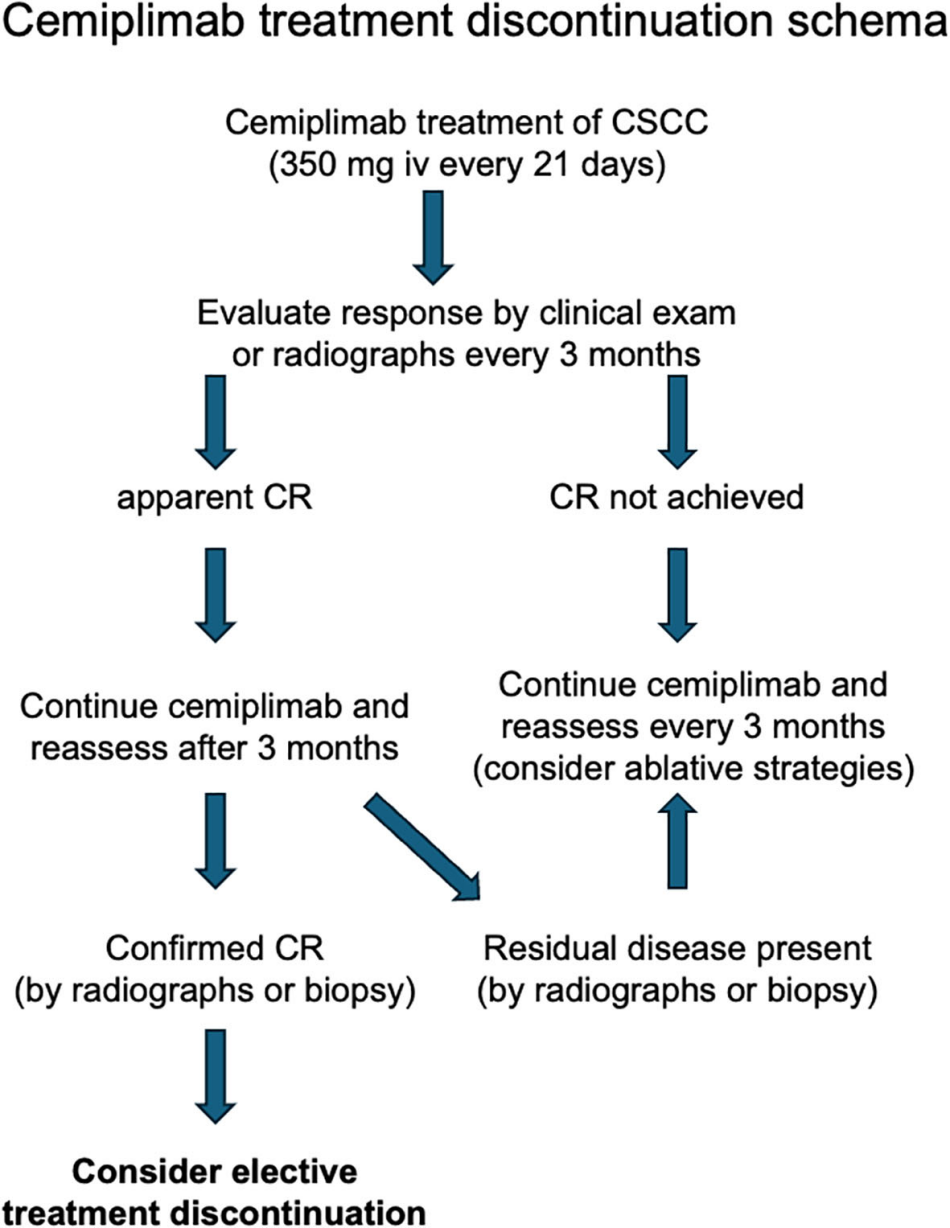
Elective treatment discontinuation schema.

Patients were considered for elective treatment discontinuation if they achieved a radiologic complete remission (CR), confirmed by two negative scans taken at least three months apart as previously described A treatment discontinuation schema is provided (**Figure 1**) (20, 21). Identification of clinical complete responses for patients with locally advanced disease that was not measurable by radiographs was challenging, as there was frequently residual abnormal scar tissue at the treatment site. For these patients, as well as patients with persistent stable radiologic abnormalities, biopsies were performed to confirm a pathologic complete remission. Patients who attained either radiologic or pathologic remission continued cemiplimab therapy for an additional 3 months, followed by a radiologic or clinical confirmation of their remission prior to discussion of elective treatment cessation.

### 2.5 Statistical analysis

Basic descriptive statistics were computed using Microsoft Excel. Progression-free survival (PFS) was assessed using the Kaplan–Meier method (25) and was defined as the time from elective treatment discontinuation to documented disease recurrence or last follow-up. Survival analyses were performed using IBM SPSS Statistics for Windows, Version 29.0 (IBM Corp., Armonk, NY). Kaplan–Meier estimates of PFS with corresponding 95% confidence intervals were calculated using Greenwood’s variance estimator. Given the limited cohort size and the occurrence of a single progression event, all analyses were descriptive in nature, and no formal comparisons between disease stage subgroups were performed. Confidence intervals were reported for the pooled PFS estimate where estimable; subgroup- and overall survival–specific confidence intervals were not reported due to the absence or rarity of events.

## 3 Results

### 3.1 Patient demographics

Twenty-one patients with metastatic or locally advanced squamous cell skin cancer were treated with single-agent cemiplimab (**Supplementary Table 1**). Fifteen of these 21 patients (71.4%) achieved a confirmed complete remission (CR) following cemiplimab treatment and underwent elective treatment discontinuation. Six patients either did not achieve a CR (2 patients), or they were lost to follow-up (4 patients). Among the 15 patients included in the analysis, 9 had locally advanced tumors, while the remaining 6 had metastatic disease (**Table 1**).

**Table 1:** Individual patient treatment and outcome.

UPN	Age	Sex	Race	Comorbidities	Dx	Primary Site	Mets	Doses	Treatment duration (mo)	Potential follow up (mo)	Toxicity	PFS (mo)	PFS from EOT (mo)	OS from EOT (mo)	Current Status
1	68	M	C	HTN, osteoarthritis	M	Unk	LN	8	4.9	38	None	44.1	39.2	39.2	NED
2	84	M	C	Osteoarthritis, HTN, prostate CA, hypothyroidism	M	scalp	In-transit	6	4.6	21.4	None	27.7	23.1	23.1	NED
3	77	M	C	Asthma, hypercholesteremia	M	Unk	LN	8	5	29	Fever/chills, rash (mildly pruritic), dizziness	37.4	32.4	32.4	NED
4	80	M	C	Arthritis, HTN, prior stroke, gout, hyperlipidemia, prostate CA	M	face	Parotid	7	6.4	6.6	None	14.3	0.9	0.9	NED
5	79	F	C	Asthma, HTN, intermittent bronchitis	LA	Scalp		10	6.7	14.3	None	21.3	14.7	14.7	NED
6	72	M	C	Arthritis, chronic bronchitis, essential HTN, gout, BPH, low testosterone	LA	Scalp	Parotid	8	7.8	23	Balance issues, muscle weakness, dizziness, asthenia	31.4	23.6	23.6	NED
7	88	M	C	None	LA	Face	Parotid	8	5.3	54.5	None	40.5	35.1	35.1	NED
8	82	M	C	Osteoarthritis, hypothyroidism, CAD, T2DM, HTN, lumbar spinal stenosis, bladder CA, hydronephrosis	LA	Scalp		12	7.7	61.1	None	53.7	46.0	51.9	DOD
9	49	M	C	HTN, COPD, GSW, alcoholism	LA	arms		4	3	52.1	None	47.7	44.7	44.7	NED
10	77	M	C	chronic hepatitis C	LA	arm		8	5.7	7.7	None	15.8	10.1	10.1	NED
11	64	F	H	HTN, T1DM, restless leg syndrome, arthritis, hypothyroidism	LA	face		6	5.1	35	Pain in tumor, anxiety, watery diarrhea with cramps, mild headache	43.6	38.6	38.6	NED
12	66	M	C	HIV, MI	LA	face		20	17.1	48	None	33	15.9	15.9	NED
13	72	M	C	BPH, hypogonadism, indolent NHL	M	Unk	LN	8	4.8	49.8	None	55.9	51.1	51.1	NED
14	76	M	C	Crohn’s disease	M	Parotid	Parotid	8	5.5	32	None	10.1	4.6	4.6	NED
15	80	M	C	Stroke, HTN, gout	LA	Ear		8	4.8	43	None	40.8	36.0	36.0	NED

UPN, unique patient number; M, male; F, female; C, Caucasian; H, Hispanic; M, metastatic; LA, locally advanced; Unk, unknown primary site; Mets, metastases; LN, Lymph node metastases; HTN, hypertension; T2DM, type 2 diabetes mellitus; BPH, benign prostatic hypertrophy, CLL, chronic lymphocytic leukemia; CSCC, cutaneous squamous cell carcinoma; CAD, coronary artery disease; GSW, gunshot wound; NHL, non-Hodgkin’s lymphoma; T1DM, type 1 diabetes mellitus; HIV, human immunodeficiency virus; MI, myocardial infarction; COPD, chronic obstructive pulmonary disease; OR, objective response; PFS, progression-free survival from treatment start; PFS from EOT, progression-free survival from end of therapy; OS, overall survival from treatment start.

The median age of these 15 patients was 77.9 ± 9.7 years at the start of cemiplimab treatment, underscoring the advanced age of the population that develops keratinocyte carcinomas. There were 13 men and 2 women. All patients were Caucasian. Five of the 15 patients had underlying conditions that may have contributed to altered immunologic function and predisposed to CSCC development. These underlying conditions included chronic lymphocytic leukemia (Rai stage 0)(1 patient), previously treated chronic hepatitis C (1), HIV infection (1), CD5+ non-Hodgkin lymphoma (1), and ongoing treatment with azathioprine and ustekinumab for colitis (1). Most of our patients (11 out of 15) had undergone prior surgical resection, while 4 patients had no prior surgery. 3 out of 15 patients had prior radiotherapy. Clinical data describing all 21 treated patients is provided (**Supplementary Table 1**).

### 3.2 Cemiplimab therapy

The median duration of cemiplimab treatment prior to treatment discontinuation was 5.3 ± 3.7 months, with a median of 8.0 ± 3.6 doses administered. Median potential follow-up was 32 ± 17.3 months (**Table 1**).

### 3.3 Outcome of elective treatment discontinuation

Among 15 CSCC patients who discontinued cemiplimab in confirmed CR, only one patient ever developed a recurrence, both locally on the scalp and with metastases to the skull. Eventually this patient died of his disease. This recurrence occurred 46 months following treatment discontinuation. One additional patient resumed cemiplimab therapy for a new locally advanced basal cell carcinoma (BCC) at a distinct anatomic location.

**Figure 2:**
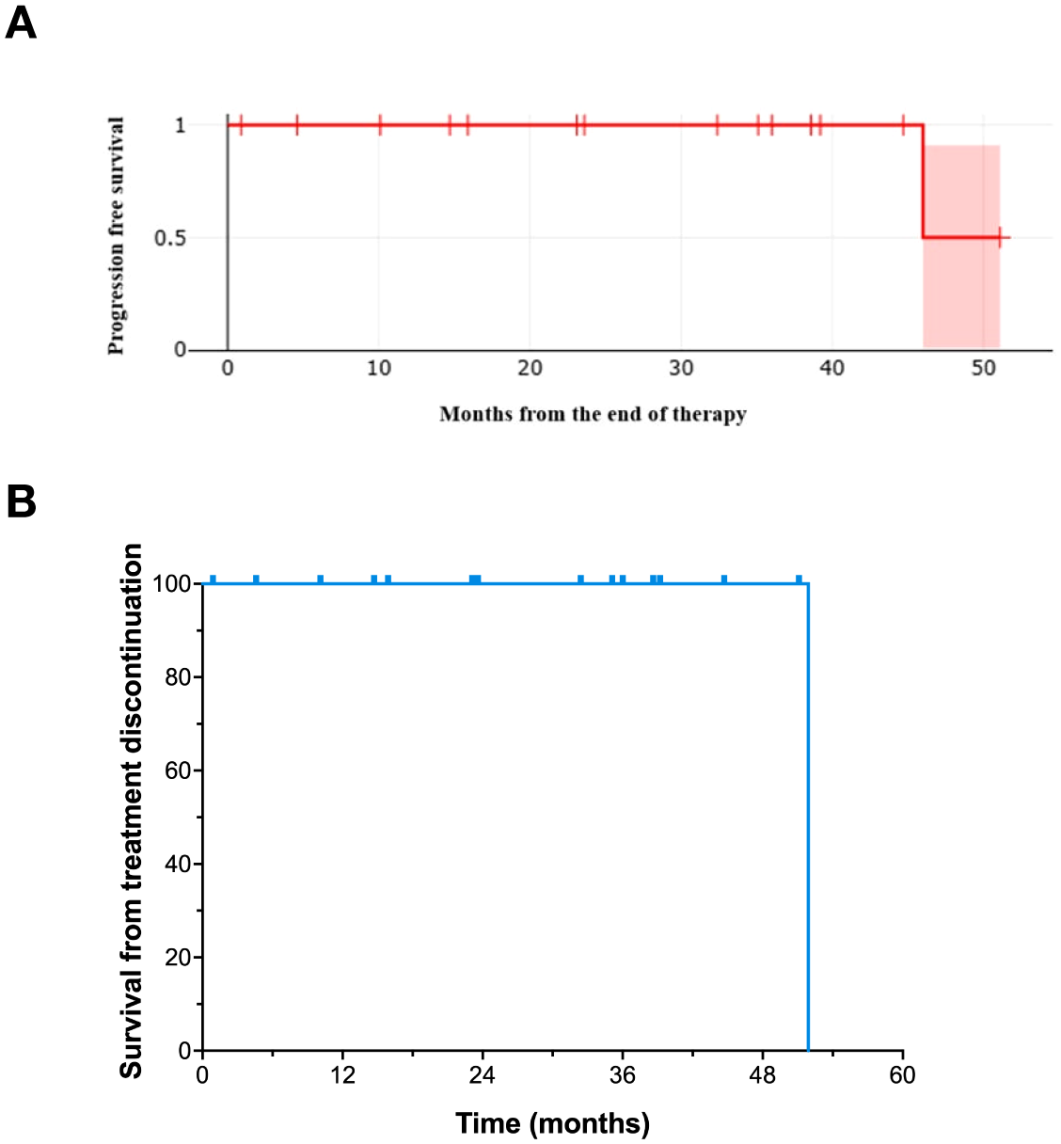
Progression-free survival of patients who underwent elective treatment discontinuation. **(A)** Progression-free survival from the time of elective treatment discontinuation (with 95% confidence intervals). **(B)** Overall survival from the time of elective treatment discontinuation.

Median progression-free survival (PFS) from the start of therapy in this cohort of complete response patients was 54.8 months. Median progression-free survival was also analyzed from the time of elective treatment discontinuation (**Figure 2A**). Estimated median progression-free survival from the end of treatment was at least 46 months. Overall survival (OS) was 100% at 4 years (**Figure 2B**).

**Figure 3:**
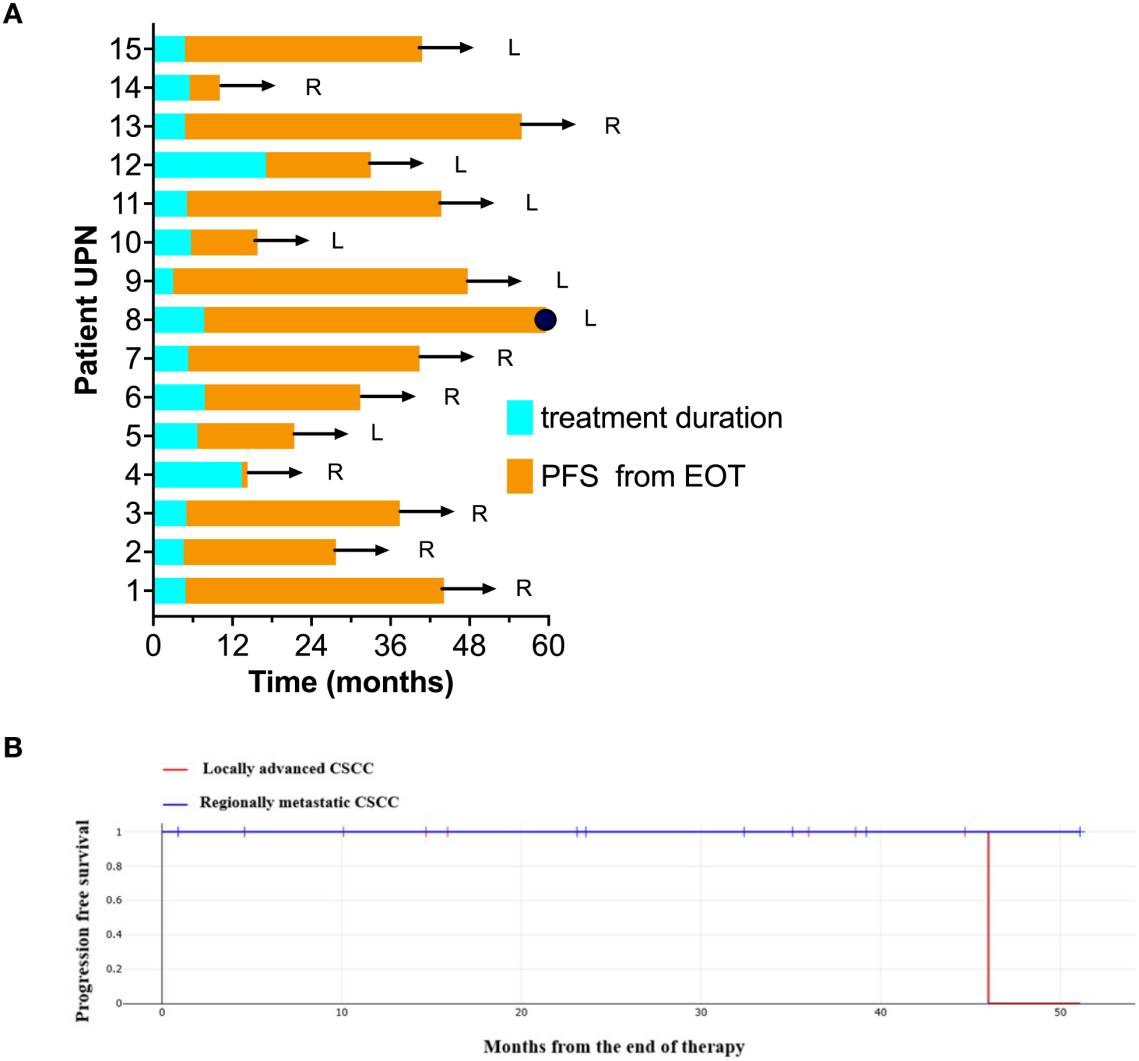
**(A)** A “swim lane” plot shows the duration of cemiplimab therapy in individual patients (blue). The length of follow-up after elective treatment discontinuation is also indicated (orange). Arrows represent patients in an ongoing unmaintained complete remission. A dot represents tumor progression in one patient. Patients treated for locally advanced disease are indicated (L), as are those with lymph node metastases or in-transit metastases (R). **(B)** Progression-free survival for patients with locally advanced or regionally metastatic CSCC.

One patient relapsed at 46 months from treatment discontinuation and died of metastatic disease at 51.9 months following elective treatment discontinuation. This patient had bone (skull) involvement at diagnosis. The duration of cemiplimab therapy and the length of subsequent follow-up following elective treatment discontinuation for individual patients is shown in a “swim lane” plot (**Figure 3A**).

To address potential heterogeneity by disease stage, progression-free survival from end of therapy was also examined in stage-stratified descriptive analyses. Among the 15 patients who achieved complete remission and underwent elective treatment discontinuation, 9 had locally advanced disease and 6 had regionally metastatic disease. These outcomes are shown (**Figure 3B**). No early or intermediate recurrences were observed in either disease stage subgroup following treatment discontinuation. At 46 months following treatment discontinuation, the Kaplan–Meier estimate of progression-free survival was 50% (95% CI: 15%–77%), with wide confidence intervals reflecting the limited number of events. For the locally advanced subgroup, the occurrence of only a single event in a patient with locally advanced CSCC resulted in a non-informative confidence interval, and subgroup analyses were therefore presented descriptively. In the regionally metastatic subgroup, no progression events were observed, and confidence intervals were not estimable, as all patients were censored.

### 3.4 Treatment related toxicity

Cemiplimab-related toxicities were readily manageable in the outpatient setting. These toxicities were all CTCAE Grade 1-2. These included fever/chills, pruritic rash, dizziness, balance issues, muscle weakness, asthenia, pain at the tumor site, diarrhea, and headache (**Table 1**). No patients in our series required hospitalization and there were no treatment related fatalities.

## 4 Discussion

The true incidence of squamous cell carcinoma in the United States is difficult to determine, due to lack of reporting requirements. It is estimated that there are over 1 million new cases per year (26). A retrospective US cohort study in patients with CSCC, suggested a local recurrence risk of 4.6%, a nodal metastasis rate of 3.7%, with distant metastasis developing in 0.4% (27). The resulting disease-specific death rate was estimated to be 2.1% (27). Thus, as many as 20-40,000 patients/year may require treatment for locally advanced or metastatic CSCC. In the past, these patients were predominantly treated with surgery or radiotherapy.

More recently, the development of the PD-1 monoclonal antibodies cemiplimab and pembrolizumab have represented a major therapeutic breakthrough for treatment of advanced CSCC (8, 9). Clinical trials demonstrated that these PD-1-directed monoclonal antibodies can mediate rapid and deep tumor responses in a high percentage of patients. The median time to response was quite rapid with both agents (1.9 months with cemiplimab and 2.25 months with pembrolizumab) (9). Both agents appear to induce durable progression-free survival in approximately 50% of treated patients (8, 9). In our recently published single-institutional experience we found that 61.1% of treated CSCC patients achieved a clinical or pathologic complete remission (13). Our institutional results may have been better than those in previously studies due to the use of biopsies rather than medical photography to assess responses.

High clinical response rates following ICI treatment of CSCC may be attributed to several tumor-specific factors. These include elevated levels of PD-L1 expression, especially in advanced CSCC lesions, which can inhibit T cell cytotoxicity via the lymphocyte inhibitory molecule PD-1 on cytotoxic T cells (28, 29). CSCC tumors frequently have an elevated tumor mutation burden due to UV-induced damage (30, 31). This may promote immune recognition through the increased number of mutated tumor antigens presented to the immune system. The involvement of cutaneous viruses in the pathogenesis of CSCC has also been proposed (32). This leads to the possibility of enhanced immune recognition of viral antigens by T cells (33).

The optimal treatment duration required to maintain ICI-induced remissions before elective treatment discontinuation can safely be considered remains uncertain (34). In clinical trials of cemiplimab and pembrolizumab, these patients were generally treated for an arbitrary length of time (at least 1–2 years) in the absence of major immune related adverse events (IRAE) (8, 14). Currently, there is no standardized guideline to guide duration of cemiplimab treatment.

There are potential disadvantages related to prolonged cemiplimab administration beyond complete remission. While most immune related adverse events have an acute onset, late or chronic toxicities related to prolonged therapy have been reported (15, 16). Rheumatologic as well as endocrine toxicities seem to be the most common delayed toxicities, although toxicities involving other organ systems have also been reported. Ongoing treatment can also add significantly to medical expenses for patients and payers (17, 18). Lengthy ICI treatment can have an adverse effect on patient quality of life (19). In addition, there is a concern that long-term ICI therapy could trigger pathologic conditions associated with systemic inflammatory responses, such as accelerated atherosclerosis (35).

We have developed and tested a treatment discontinuation strategy in ICI treated melanoma patients that appears to result in a low relapse rate (20). This strategy has also been effective in other tumor types (21). We performed a retrospective evaluation of elective treatment discontinuation in cemiplimab-treated CSCC patients.

In our current patient series, 15 of 21 cemiplimab treated patients with locally advanced or metastatic CSCC achieved a complete remission (71.4%). These 15 patients were considered for potential elective treatment discontinuation. The median treatment duration to achieve a confirmed clinical complete remission was only 5.3 ± 3.7 months, after a median of 8.0 ± 3.6 doses of cemiplimab. The median progression-free survival after treatment cessation was over 48.5 months. Fourteen out of fifteen patients who underwent elective treatment discontinuation currently remain alive and free of recurrence.

A unique challenge in the CSCC patient population is that some patients do not have radiologically measurable disease. Clinical response is usually apparent due to a decrease in the peripheral rim of tumor tissue and healing of pathologic ulcerations. In early clinical trials, medical photography was used to evaluate treatment responses. This likely resulted in misclassification of complete responses as partial responses due to persistent scar tissue at the treated site. In these indeterminate situations, we have performed biopsies of abnormal-appearing areas to confirm a pathologic complete remission prior to treatment discontinuation. In patients with persistent viable tumor, treatment was continued.

There is relatively little prior published information concerning the safety of early treatment discontinuation. A 2024 report by Boutros et al. found that among 17 patients who discontinued cemiplimab early either due to toxicity or physician choice, there were no relapses if patients had achieved a complete remission or partial response (36). A second study by Bailly-Caille et al. (2024) reported 14 cemiplimab treated patients whose treatment was discontinued, due to a loss of reimbursement (37). Of these 14 patients, 9 patients had achieved a complete response, 3 a partial response, one patient had a stable disease, and one patient had a progressive disease. Unlike our patient series, 5 had received concomitant cemiplimab and radiotherapy. At 12 months following cemiplimab discontinuation, the 8 CR patients maintained a persistent complete response, but one of 3 patients with a partial response progressed. At 24 months after stopping cemiplimab 6 of the 8 patients had an ongoing complete response: One patient was lost to follow-up, and one patient had developed progressive disease. Neither of these reports identified specific criteria for planned elective treatment discontinuation. While our radiologic or pathologically established complete remission rate appears high compared to initial studies that employed medical photography to assess response, our results are similar to other more recent publications. For example, in a retrospective review of CSCC patients undergoing elective treatment discontinuation following either pembrolizumab or cemiplimab induced remissions, Averbuch et al. reported a 73% overall response rate (38). Following a median follow-up of 29.9 months, 60% of their patients in the treatment-break group remained progression-free. Similarly, in a neoadjuvant pembrolizumab treatment trial, Ladwa et al. reported a complete remission rate of 63%, which allowed de-escalation of therapy, omitting planned surgery and radiotherapy. None of these patients recurred with a median follow-up of 18 months (11).

While our study provides valuable insights, several limitations should be considered. First, data were collected via a retrospective review of existing records rather than through a controlled, prospective protocol. This approach may, therefore, be affected by unmeasured confounding factors, which might contribute to the residual confounding bias. Second, the relatively small patient cohort may affect the generalizability of the findings to broader patient populations. In addition, the limited number of patients within each disease stage subgroup precludes definitive conclusions regarding stage-specific recurrence risk. Potential referral bias could also have impacted our results. Accordingly, these findings should be interpreted as descriptive and hypothesis-generating and warrant confirmation in larger prospective studies.

In conclusion, our study suggests that elective discontinuation of cemiplimab after achieving complete remission is feasible and safe. A complete response by radiographs or pathology was often obtained after just 4 doses of cemiplimab (3 months). Including continuation of therapy for 4 additional doses of cemiplimab after initial detection of a complete response, the median treatment duration in our patients was less than 6 months. Our findings suggest that most complete responses achieved in CSCC patients are rapidly achieved and are quite durable. Only one patient experienced a late recurrence after 48 months. Thus, this treatment discontinuation protocol may be useful in reducing the risk of late onset ICI toxicity and treatment-related costs. While these results are encouraging, further prospective evaluation of treatment discontinuation is needed to define the optimal duration of cemiplimab therapy.

## Data Availability

The raw data supporting the conclusions of this article will be made available by the authors, without undue reservation.
